# Porous CeO_2_/CuO Heterostructure for Efficient Hydrogen Evolution Reaction in an Acidic Medium

**DOI:** 10.1002/open.202500115

**Published:** 2025-06-05

**Authors:** Binod Raj KC, Samira Munkaila, Bishnu Prasad Bastakoti

**Affiliations:** ^1^ Department of Chemistry North Carolina Agricultural and Technical State University 1601 E. Market St Greensboro NC 27411 USA

**Keywords:** CeO_2_/CuO, electrocatalysts, heterostructures, hydrogen evolution reactions, oxygen vacancies

## Abstract

A composite electrocatalyst CeO_2_/CuO is fabricated for the hydrogen evolution reaction (HER) via a simple, facile, one‐step hydrothermal method, using F‐127 as a template and structure directing agent. The prepared composite CeO_2_/CuO shows top‐notch HER activities with a small overpotential of 98 mV at 10 mA cm^−2^ and just 160 mV overpotential to attain a current density of 50 mA cm^−2^ with good stability for 20 h in an acidic medium. The enhanced catalytic activities of CeO_2_/CuO nanocomposite are attributed to their synergetic interface interaction, which lead to improved conductivity, reactive sites, and oxygen vacancies in their lattices. This study aims to advance the development of earth‐abundant transition metal oxides–based electrocatalysts as economical, durable, and efficient HER electrocatalysts to replace noble metal–based materials in the future.

## Graphical abstract

Mesoporous CeO_2_/CuO nanocomposite is synthesized via a facile and economical one‐pot hydrothermal method. This synthetic approach enables the formation of a well‐defined heterostructure with a highly porous architecture, providing a large specific surface area and abundant active sites for electrocatalytic reactions in hydrogen evolution applications for sustainable energy systems.

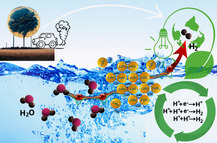

## 1 Introduction

There is an urgent need for the development of sustainable and renewable energy technologies in present scenarios of the world to address significant concerns like growing demand for energy due to thrive in population, quick exhaustion of carbonaceous fuels, variation in weather patterns, global temperature rising, and related environmental deterioration as the consequence of excessive use of natural gas.^[^ ^1^, ^2^, ^3^, ^4^, ^5^ ^]^ In recent decades, different techniques like hydro, geothermal, wind, biomass, solar power, etc., have been applied to overcome the energy crisis.^[^ ^6^ ^]^ In the front line, green hydrogen fuel is cost‐effective, having superb specific energy density (143 MJ kg^−1^), sustainable as well as pollution‐free/clean, and is a potential contender for an energy carrier which can replace fossil fuels and address environmental concerns in the future.^[^ ^7^, ^8^, ^9^ ^]^ Among multiple methods available, electrocatalytic decomposition of water is one of the rising direct, clean, appealing, propitious, and commercial technologies for producing green hydrogen as a source of fuel with a zero carbon footprint in the environment.^[^ ^10^, ^11^, ^12^, ^13^ ^]^ This method has the advantage of storing chemical energy produced by electricity, solar power, or both.^[^ ^14^ ^]^

A substantial attempt has been made to find an efficient and powerful hydrogen evolution reaction (HER) electrocatalyst with a lower energy barrier for water splitting and the ability to boost the unfavorable higher overpotential.^[^ ^15^ ^]^ Several noble metal‐based materials, such as Pt‐based materials, are regarded as the most effective electrocatalysts for HER.^[^ ^16^, ^17^, ^18^ ^]^ Nonetheless, they are scarce and costly, restricting their practical use.^[^ ^17^ ^]^ Hence, it is crucial to design a cost‐effective, durable, earth‐abundant, and efficient water‐splitting electrocatalyst to replace the noble metal–based catalyst.^[^ ^17^, ^19^ ^]^ Bimetallic transition oxides have demonstrated significant potential in electrochemical activity because of their propensity to tackle variable oxidation states that accelerate HER catalysis by utilizing interactions between two cations. As a result, rationally designing bimetallic oxide electrocatalysts can expedite the adsorption, conversion, and desorption of intermediates at the time of electrolysis.^[^ ^20^, ^21^ ^]^ Copper is a multivalent, environmentally benign transition metal having rich redox properties and an abundant supply with the most stable copper oxide (CuO, tenorite) phase.^[^ ^22^, ^23^ ^]^ Cu‐based oxides are considered effective oxygen carriers because of their reversible conversion between Cu^+^ and Cu^2+^ ions, allowing control over neighboring atoms’ electronic conditions.^[^ ^22^ ^]^ In addition, Cu‐containing oxides are efficient for HER due to their low energy barrier for hydrogen adsorption.^[^ ^24^ ^]^ In contrast, CeO_2_ is also investigated as a potential HER catalyst due to its special physicochemical properties, such as superior redox propensity and rich oxygen vacancy defects, as well as its reasonable chemical and mechanical stability.^[^ ^25^, ^26^ ^]^ Highly active redox couples (Ce^3+^ and Ce^4+^) within CeO_2_ and the surface oxygen ion exchange make CeO_2_ a potential candidate for significant electrochemical activities, as well as creating profound interactions with other materials as a matrix.^[^ ^26^, ^27^ ^]^ Combining CeO_2_ with CuO can be a promising approach for optimizing the HER of CeO_2_ through a synergistic coupling effect.

Herein, we demonstrate the fabrication of a porous CeO_2_/CuO heterostructure by a straightforward, facile, and economical one‐pot hydrothermal method. The prepared composite material displayed excellent HER activity and durability in an acidic medium. The metal oxides used to prepare a composite of CeO_2_/CuO are naturally abundant transition element sources. The synergistic effect with its counterparts, presence of oxygen vacancies, enhanced conductivity, and improved adsorption affinity for hydrogen intermediates by bringing down the energy obstruction are primary reasons for superior performance of this electrocatalyst in HER.

## 2 Results and Discussion

**Figure 1:**
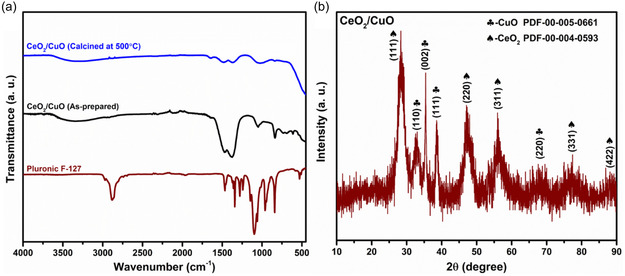
a) FTIR spectra of Pluronic F‐127, CeO_2_/CuO as prepared sample and CeO_2_/CuO calcined at 500 °C. b) XRD pattern of CeO_2_/CuO‐0.5 composite after calcination at 500 °C.

**Figure** **1**a depicts how transformations occur in the peak positions of the F‐127, uncalcined CeO_2_/CuO, and CeO_2_/CuO calcined at 500 °C. When cerium and copper sources were added to the host F‐127 polymer matrix, the spectra obtained in the prepared sample of CeO_2_/CuO showed a shifting of Fourier transform infrared spectroscopy (FTIR) signals. The stretching band at 400 cm^−1^ corresponds to the Ce—O bond, while the band in connection with 622 cm^−1^ represents the presence of cuprous oxide.^[^ ^28^ ^]^ Pluronic F‐127 [poly(ethylene oxide)–poly(propylene oxide)–poly(ethylene oxide)] (PEO–PPO–PEO) behaves as an appropriate amphiphilic copolymer in aqueous solution as a structure‐directing agent through hydrogen bonding interactions. The changes in peaks at the region between 500 and 1500 cm^−1^ in as‐prepared sample revealed intense interactions between polymer matrix and metals precursors (Ce^3+^ and Cu^2+^). The existence of the PEO chain in F‐127 is the leading reaction site for the interaction of Ce and Cu ions.^[^ ^29^ ^]^ After annealing, the characteristic O—H vibration during stretching of hydroxides and remaining water molecules, which belong to the spectrum close to 3450 cm^−1^,^[^ ^30^ ^]^ drastically decreased. The prominent peak at 1483 cm^−1^ can be credited to the oscillating methylene C—H bond, while a typical substantial peak that occurs at ≈2300 cm^−1^ is assigned to the adsorbed carbon dioxide. These peaks eventually vanished overtime as a consequence of the removal of the polymer molecules from the resulting binary oxide after annealing.^[^ ^31^ ^]^

Powder X‐ray diffraction (XRD) analysis was done to examine the crystalline structure and phase purity of as prepared and calcined samples. The sample synthesized by using only Cu and Ce precursors showed the XRD pattern (Figure S1a, Supporting Information), which is well‐matched with PDF File #00‐005‐0661 of CuO and CeO_2_ diffraction peaks (Figure S1b, Supporting Information), which were marked based on the File #00‐004‐0593. The XRD pattern of CeO_2_/CuO‐0.5 heterostructure annealed at 500 °C shows characteristic diffraction peaks from CeO_2_ and CuO (Figure 1b). These findings confirm the formation of two‐phase CeO_2_/CuO heterostructure composites. The XRD pattern of the as‐prepared samples is shown in Figure S1c, Supporting Information. Calcination removes organic moieties and induces crystallinity. Thermogravimetric analysis (TGA) was conducted in ambient conditions to study the thermal behavior of the as‐prepared sample (Figure S1d, Supporting Information). This depicts the thermally stable properties of the catalyst, affirming the appropriate calcination temperature conditions for highly effective catalysis without an aberration in the structure. The TGA curve revealed a two‐stage weight loss process; the first step is associated with removing water molecules, and the second steep curve at about 435 °C is related to the decomposition of the polymer.^[^ ^32^, ^33^ ^]^ The thermal endurance of the nanocomposite has been proven with about a 20% drop in weight up to 750 °C.

**Figure 2:**
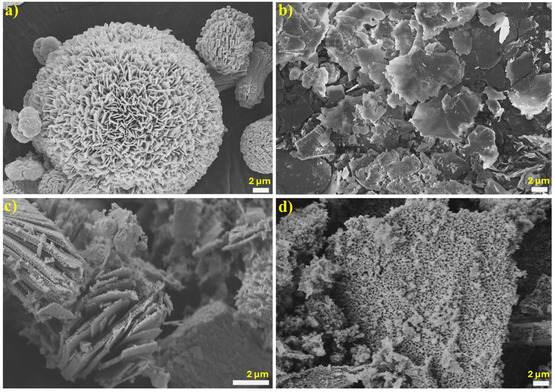
FESEM images of a) CuO, b) CeO_2_, c) CeO_2_/CuO‐0.5, and d) CeO_2_/CuO‐1 calcined at 500 °C.

**Figure 3:**
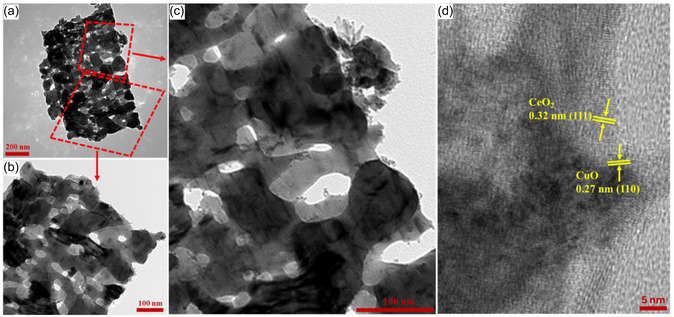
a,b) TEM images, c) HRTEM of composite, and d) HRTEM showing lattice spacings of CeO_2_ and CuO of CeO_2_/CuO‐0.5.

The copper oxide primarily displayed an assembly of flat rods integrated and clumped together (**Figure** **2**a), whereas the cerium oxide showcased a lamina‐like morphology (Figure 2b). The composite CeO_2_/CuO (Figure 2c,d) displayed a loosely bonded, porous, inter‐packed lamina‐like structure. The surface morphology of the CeO_2_/CuO‐0.5 composite without F‐127 is shown in Figure S2, Supporting Information, with irregular morphology, where porosity was not observed. Energy‐dispersive X‐ray spectroscopy (EDX) mapping was used to evaluate the most effective ratio (CeO_2_/CuO‐0.5) for homogeneous intermetallic proportions of weights for Ce, Cu, and O elemental compositions (Figure S3, Supporting Information). Furthermore, transmission electron microscopy (TEM) analysis was performed to study the nanostructure morphology of CeO2/CuO‐0.5, and the results are presented in **Figure** **3**a–c. The low‐magnification TEM images of optimal hybrid catalyst ratio displayed a mesoporous inter‐packed flake‐like structure. High‐resolution TEM (HRTEM) of CeO_2_/CuO‐0.5 displaying the lattice spacing of CeO_2_ and CuO is shown in Figure 3d. The mesoporosity on CeO_2_/CuO‐0.5 composite facilitates a more accessible diffusion of ions and gas molecules in catalytic reaction.^[^ ^34^, ^35^ ^]^

**Figure 4:**
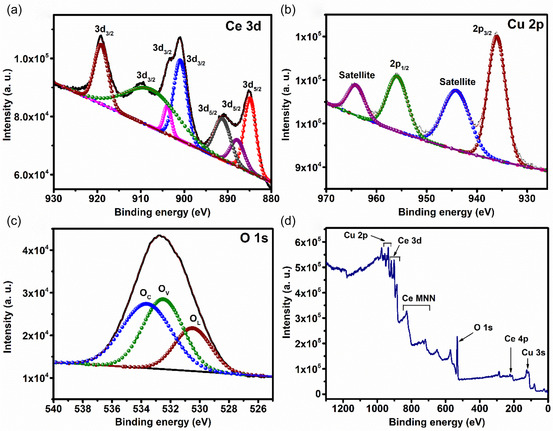
XPS spectra of a) Ce 3d, b) Cu 2p, c) O1s, and d) survey spectra of CeO_2_/CuO‐0.5 L.

To analyze the surface elemental constitution and valence state of calcined CeO_2_/CuO, X‐ray photoelectron spectroscopy (XPS) was conducted, as displayed in **Figure** **4**a–d. The survey spectrum of calcined CeO_2_/CuO (Figure 4d) manifests the presence of a series of peaks corresponding to Cu 2p, Ce 3 d, and O 1s, demonstrating the successful fabrication of the composite material. Deconvoluted Ce 3 d core‐level spectra ranging from 880 to 930 eV manifest the existence of two sets of spin–orbit split doublets of Ce 3d_5/2_ and Ce 3d_3/2_, supporting the simultaneous presence of both Ce^3+^ and Ce^4+^ ions.^[^ ^36^, ^37^, ^38^ ^]^ The characteristic peaks located at 884.88, 891.22, 900.90, 907.53, and 919.10 eV correspond to Ce^4+^, while representative peaks at 887.78 and 903.84 eV indicate the presence of Ce^3+^.^[^ ^39^ ^]^ Ce^3+^ and Ce^4+^ in the sample revealed the redox nature of the mixed oxide catalysts.^[^ ^40^, ^41^ ^]^ When Ce^4+^ is reduced to Ce^3+^, oxygen is released, increasing oxygen concentration on the catalyst surface.^[^ ^15^, ^42^ ^]^ Copper showed a Cu 2p_3/2_ peak corresponding to binding energy at 936.05 eV, along with one satellite peak located at 944.09 eV and Cu 2p_1/2_ at 955.67 eV, along with one prominent satellite peak (Figure 4b).^[^ ^22^, ^41^, ^43^ ^]^ These results align with the Cu 2p binding energy for Cu^2+^ in mixed oxide samples.^[^ ^43^, ^44^ ^]^ The composite's O 1s spectrum is deconvoluted into three peaks (Figure 4c) corresponding to binding energy 530.48, 532.50, and 533.62 eV that are associated with O^2−^ species in lattice (O_L_) coordinated with Ce^4+^, oxygen defects/vacancy (O_V_) on surface, and chemisorbed oxygen species (O_C_), respectively.^[^ ^45^, ^46^ ^]^

**Figure 5:**
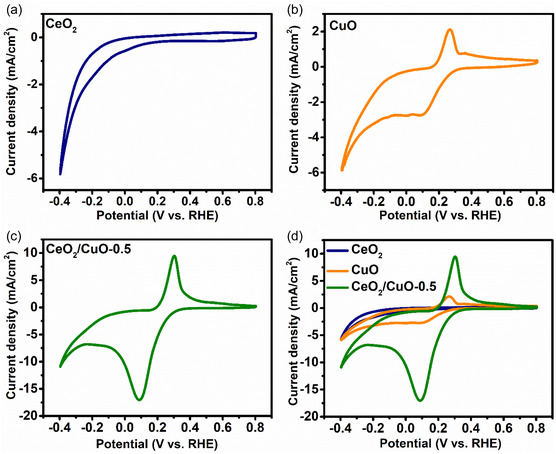
Cyclic voltammograms of a) CeO_2_, b) CuO, c) CeO_2_/CuO‐0.5, and d) comparison of all of them at a scan rate of 40 mVs^−1^.

The electrocatalytic performance and surface oxidation states of samples were evaluated using cyclic voltammetry (CV) measurements ranging from −0.4 to 0.8 V versus reversible hydrogen electrode (RHE). Cyclic voltammograms of different samples and their comparison at a constant sweeping rate of 40 mVs^−1^ are displayed in **Figure** **5** and S4, Supporting Information. The CV profile of CeO_2_/CuO‐0.5 exhibits a rectangular‐shaped curve with distinct anodic and cathodic peaks, indicating the impact of redox behavior in electrodes.^[^ ^47^ ^]^ Compared with CV profiles of other samples (CeO_2_, CuO, and CeO_2_/CuO‐1), the current density values and their associated area were significantly higher in CeO_2_/CuO‐0.5. This result shows better conductivity of the sample for HER.^[^ ^48^ ^]^ The intensive activity could be pointed out to their proportionate count of Cu^+^/Ce^3+^ redox pairs, the presence of a greater number of corresponding defects and oxygen vacancies, as well as synergistic effect between CeO_2_ and CuO.^[^ ^49^ ^]^

**Figure 6:**
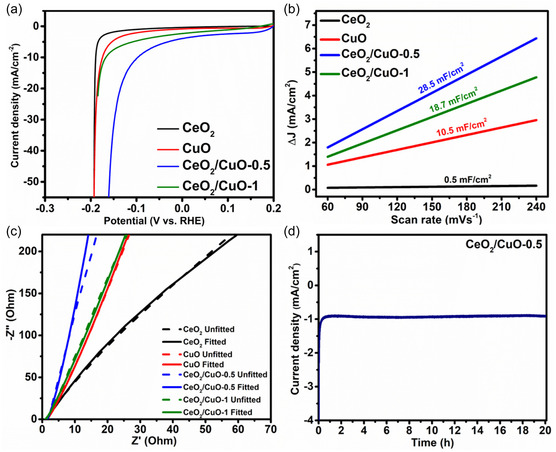
a) Linear sweep voltammetry, b) electrochemical active surface area, c) Nyquist plot for all samples, and d) chronoamperometric curve for CeO_2_/CuO‐0.5 at −0.0971 V versus RHE.

All samples were tested for electrochemical HER activity using linear sweep voltammetry in 1M H_2_SO_4_ at voltages that go from −0.3 to 0.2 V versus RHE (**Figure** **6**a). There is a significant rise in current density at −0.1 to −0.2 V (vs. RHE), indicating the HER activity of composite samples as displayed by polarization curve. The HER activity of samples can be determined by the overpotential (η_10_) necessary to generate 10 mA cm^−2^ current density, that is comparable to 10% solar energy transformation rate.^[^ ^10^, ^50^, ^51^ ^]^ Composites have lower η_10_ values than pure CeO_2_ and CuO, indicating superior HER performance of composites compared to their counterparts. When HER activity of all the samples (CeO_2_, CuO, CeO_2_/CuO‐0.5, and CeO_2_/CuO‐1) was compared, the lowest η_10_ was found to be associated with CeO_2_/CuO‐0.5 (n_10_ = 98 mV) indicating best composite for HER compared to others (CeO_2_/CuO‐1 = 167, CuO = 175, CeO_2_ = 190 mV). Composite CeO_2_/CuO‐0.5 necessitates an overpotential of 160 mV to attain a current density of 50 mA cm^−2^. The reason for the lowest overpotential for this composite ratio might be attributed to the availability of a large number of protons in an acidic medium, which promoted HER catalysis.^[^ ^52^, ^53^ ^]^ In acidic electrolytes, HER is a crucial step in the electrochemical water‐splitting process and involves generating hydrogen by electrochemically reducing H^+^ ions.^[^ ^54^, ^55^ ^]^

Electrochemically active surface area (ECSA) is crucial in identifying catalyst‐active sites. ECSA of all the prepared samples (Figure 6b) was estimated by measuring the CV polarization curve in the non‐Faradaic region at different scan rates (Figure S5, Supporting Information). This estimated surface area is proportionate to electrical double‐layer capacitances (C_dl_) measured at the electrode–electrolyte interface.^[^ ^56^, ^57^ ^]^ Table S1, Supporting Information, compares the C_dl_ and ECSA values of all the prepared samples. The calculated C_dl_ and ECSA for the CeO_2_/CuO‐0.5 sample are 28.5 mF cm^−2^ and 712.5 cm^2^, respectively, the highest values among all the samples. This finding reveals the efficient catalytic activity of CeO_2_/CuO‐0.5 toward HER. When it comes to evaluating the electrocatalytic reactions, transmission of charge at the electrode/electrolyte surface is the next crucial factor to be considered.^[^ ^58^ ^]^ Electrochemical impedance spectroscopy (EIS) analysis was performed at a frequency varying from 200 kHz to 100 mHz to investigate the charge‐transfer activity and capacitive behavior of electrocatalysts. Figure 6c depicts the impedance plot of different samples, which is also known as the Nyquist plot. The CeO_2_/CuO‐0.5 sample exhibits lower contact resistance (R_ct_ = 0.1148 ohm) as well as superior capacitive value (C_dl_ = 0.343×10^−3^ F) as indicated by its steeper plot as compared to other samples (CeO_2_, CuO, and CeO_2_/CuO‐1). Lower *R*_ct_ values can be attributed to increased oxygen vacancies of electrode materials.^[^ ^59^ ^]^ Lower *R*_ct_ speeds up the movements of charged particles at the electrode/electrolyte interface, increasing the electrocatalytic activity of the material.^[^ ^21^, ^60^ ^]^ Table S2, Supporting Information, summarizes the *R*_ct_ and *C*_dl_ values of prepared samples obtained by EIS fitting. This result revealed that CeO_2_/CuO‐0.5 has prevailing performance due to its minimal diffusion barrier and enhanced charge transfer association among its constituents, assisting in the electrochemical process. A catalyst's stability is crucial for achieving long‐term and improved catalytic properties for energy conversion and storage applications. Chronoamperometric measurements examined the catalyst's stability in 1M H_2_SO_4_ at open‐circuit potential. CeO_2_/CuO‐0.5 exhibits relatively stable catalytic behavior at −0.0971 V versus RHE for more than 20 h, as displayed in the chronoamperometric curve in Figure 6d. A comparison of our work with some CuO‐ and CeO_2_‐based materials for HER is shown in Table S3, Supporting Information.

## 3 Conclusions

In this work, we successfully synthesized CeO_2_/CuO nanocomposite via a facile and economical one‐pot hydrothermal method followed by annealing in air at 500 °C. Both metal oxides cast off in the composite are earth‐abundant transition metal oxides that can be utilized for efficient electrocatalysts for HER activities. The metal sources had substantial interaction with F‐127 and ultimately facilitated the development of a nanocomposite with excellent electrocatalytic activity and durability for HER in an acidic environment. The prepared composite CeO_2_/CuO‐0.5 requires only 98 mV of overpotential to accomplish a current density of 10 mA cm^−2^ with stability of 20 h. The enhanced electrocatalytic properties of the nanocomposite can be attributed to the low energy barrier of CuO for hydrogen adsorption, synergistic coupling between CeO_2_ and CuO, the existence of oxygen vacancies, and the presence of a high ECSA. This nanocomposite can be explored more as a non‐noble electrocatalyst for electrochemical breakdown of water as a source of renewable green energy.

## 4 Experimental Section

4.1

4.1.1

##### Materials

Cerium (III) nitrate hexahydrate (Alfa Aesar, 99.5%), copper (II) nitrate trihydrate (Thermo Scientific, 99.0%), Pluronic F‐127 (Sigma Aldrich), urea (Acros Organics, 99.0%), N‐methyl‐2‐pyrrolidone (NMP) (Alfa Aesar, 99.5%), poly(vinylidene) fluoride (PVDF), carbon black (Alfa Aesar), and sulfuric acid (Fisher chemical, 98%) were used. All the experiments were conducted using deionized water.

##### Synthesis of CeO_2_/CuO *Heterostructure*

Binary CeO_2_/CuO composite materials were synthesized using a one‐pot solvothermal approach. At first, metal precursors cerium and copper salts with weight ratios of 1:0, 0:1, 1:0.5, and 1:1, respectively, were weighed and dissolved in deionized water along with urea and Pluronic F‐127 and continuously stirred at room temperature for an hour. These proportions were later presented as CeO_2_, CuO, CeO_2_/CuO‐0.5, and CeO_2_/CuO‐1 samples. The solution was heated at 90 °C for 10 h in a Teflon‐lined stainless‐steel autoclave. The solution was left to cool for 12 h. As a result, the precipitate was washed three times with deionized water and centrifuged at 8500 rpm for 45 min. The initial precipitate obtained from the centrifugation was allowed to dry at room temperature. Finally, the dried samples were annealed at 500 °C in the air for three hours at a ramping rate of 2 °C min^−1^.

##### Catalyst Characterization

The morphology of prepared samples was investigated utilizing field‐emission scanning electron microscopy (FESEM; JEOL, JSM‐IT800). An Oxford Instrument was deployed for the FESEM elemental mapping and EDX to investigate the catalyst's chemical constitution. TEM (LIBRA 120 PLUS) was used to probe the catalyst's internal morphology and crystallinity. XRD (Rigaku, Miniflex 600) was employed to ascertain the existence of cerium oxide and copper oxide and to explore their crystal phases. FTIR of the fabricated catalyst and polymer was examined using an IRTracer‐100 FTIR spectrometer. The chemical evaluation of calcined samples was performed by XPS (Thermo Scientific ESCALAB XI‐Al Kα and 200 eV). TGA(550‐TA Instruments) was performed to investigate the thermal stability of the as‐prepared sample.

##### Electrochemical Characterization

All the electrochemical tests were carried out in a three‐electrode cell configuration in 1M H_2_SO_4_ electrolyte using a BioLogic potentiostat (VSP‐3). Platinum and Ag/AgCl (3M KCl) were used as counter and reference electrodes, respectively. All potentials were referenced to the RHE, which was calibrated by implementing the equation given below.^[^ ^61^ ^]^

(1) $$E_{\text{(vs}\text{.RHE)}}\;=\;E_{\text{(Ag/AgCl)}}\;+\;0\text{.197}\;+\;0{\text{.059 pH (1M H}}_{2}{\text{SO}}_{4}\approx 0\text{.1)}$$

Here, E_(Ag/AgCl)_ refers to potential versus the reference electrode, while 0.197 stands for the standard potential of Ag/AgCl at 25 °C.^[^ ^62^ ^]^ To prepare working electrode catalyst ink for electrochemical measurement of prepared samples, 4 mg of perfectly ground calcined electrocatalyst was mixed in 400 μL of NMP followed by adding 0.5 mg of carbon black and 0.5 mg PVDF binder. The solution was sonicated for an hour to get uniformly distributed, well‐dispersed ink. A pretreated carbon cloth was used as a substrate to make the working electrode. For pretreatment of carbon cloth, first, a 1.5 cm × 1.0 cm size of carbon cloth was cut into pieces and washed with distilled water 3 times, which was then soaked in 100 mL of 1:3%v/v concentrated HNO_3_:H_2_SO_4_ solution with heating at 60 °C for 2 h. The heated carbon cloth was allowed to cool down at room temperature, rinsed with distilled water, and dried at 60 °C for 6 h. Finally, 200 μL of homogenous sonicated catalyst ink was coated on the surface of pretreated carbon cloth on an area of 1 cm^2^ and was allowed to dry in a vacuum oven at 60 °C for 14 h.

## Conflict of Interest

The authors declare no conflict of interest.

## Author Contributions

**Binod Raj KC**: data curation (equal); formal analysis (equal); investigation (equal); methodology (equal); writing—original draft (equal); and writing—review and editing (equal). **Samira Munkaila**: data curation (equal); formal analysis (equal); investigation (equal); methodology (equal); validation (equal); visualization (equal); writing—original draft (equal); and writing—review and editing (equal). **Bishnu Prasad Bastakoti**: conceptualization (lead); funding acquisition (lead); project administration (lead); resources (lead); supervision (lead); writing—original draft (equal); and writing—review and editing (equal).

## Supporting information

Supplementary Material

## Data Availability

The data that support the findings of this study are available from the corresponding author upon reasonable request.
