# Supplementary material for: Porous CeO2/CuO Heterostructure for Efficient Hydrogen Evolution Reaction in an Acidic Medium
Source: ChemistryOpen. 2025 Jun 5;14(9):e202500115. doi: 10.1002/open.202500115 (PMC12409820; doi:10.1002/open.202500115)
Supplement: Supplementary file 1 — Supplementary Material [file OPEN-14-e202500115-s001.pdf]

Supporting Information

Porous CeO_2/CuO Heterostructure for Efficient Hydrogen Evolution Reaction in an Acidic Medium

Binod Raj KC, Samira Munkaila and Bishnu Prasad Bastakoti*

Department of Chemistry, North Carolina Agricultural and Technical State University

1601 E. Market St, Greensboro, NC 27411, USA

bpbastakoti@ncat.edu

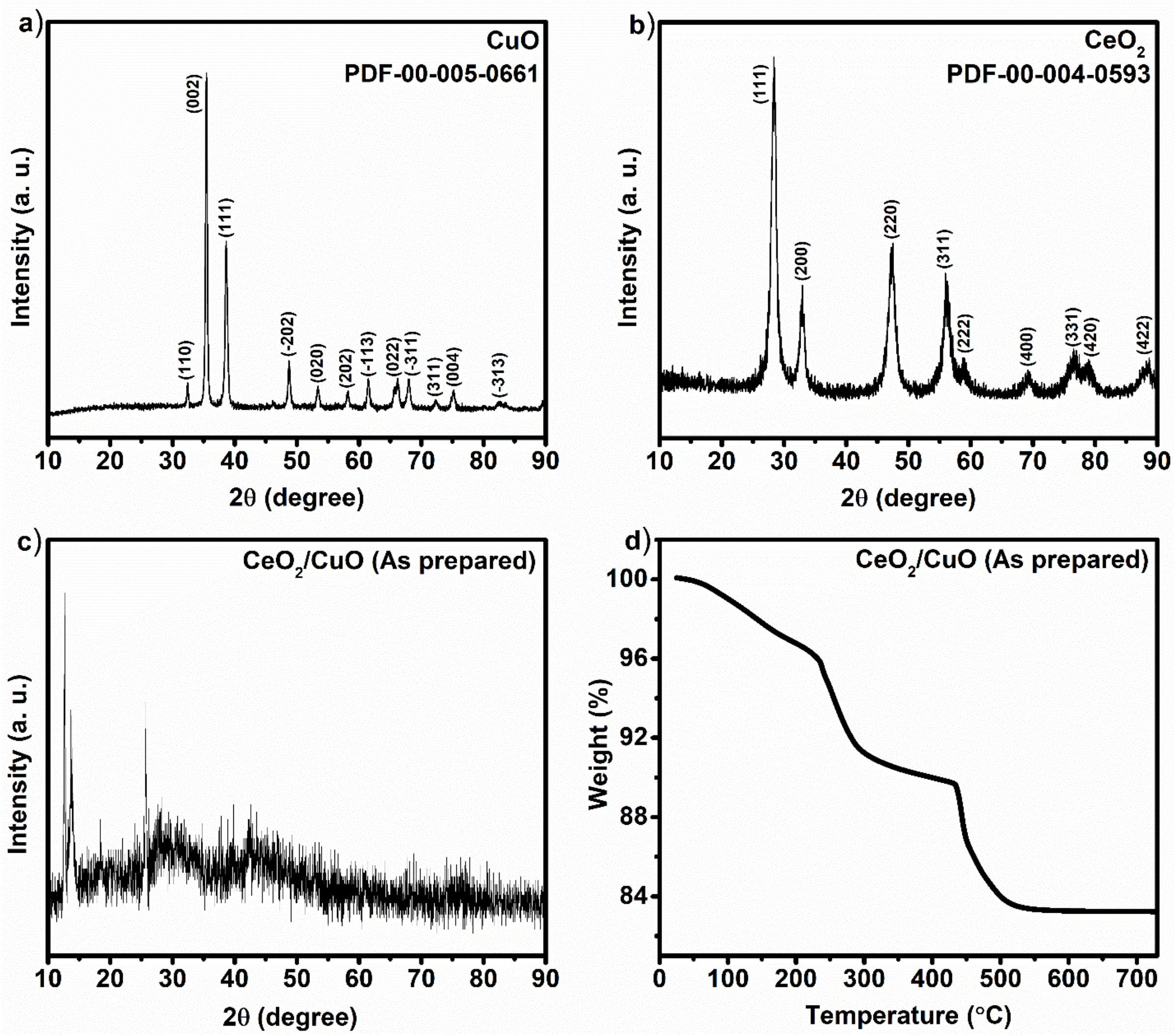

Figure S1: XRD pattern of (a) CuO calcined in air at 500 °C, (b) CeO_2 calcined in air at 500 °C, (c) CeO_2/CuO as prepared, and (d) TGA analysis of CeO_2/CuO sample in air.

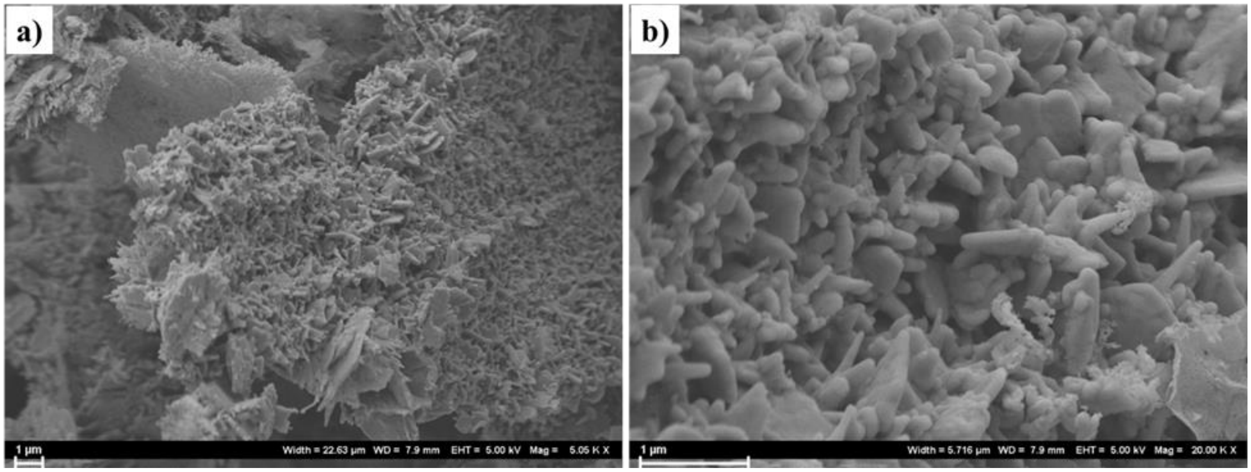

Figure S2: FESEM images of CeO_2/CuO-0.5 without F-127 calcined at 500 °C (a, b).

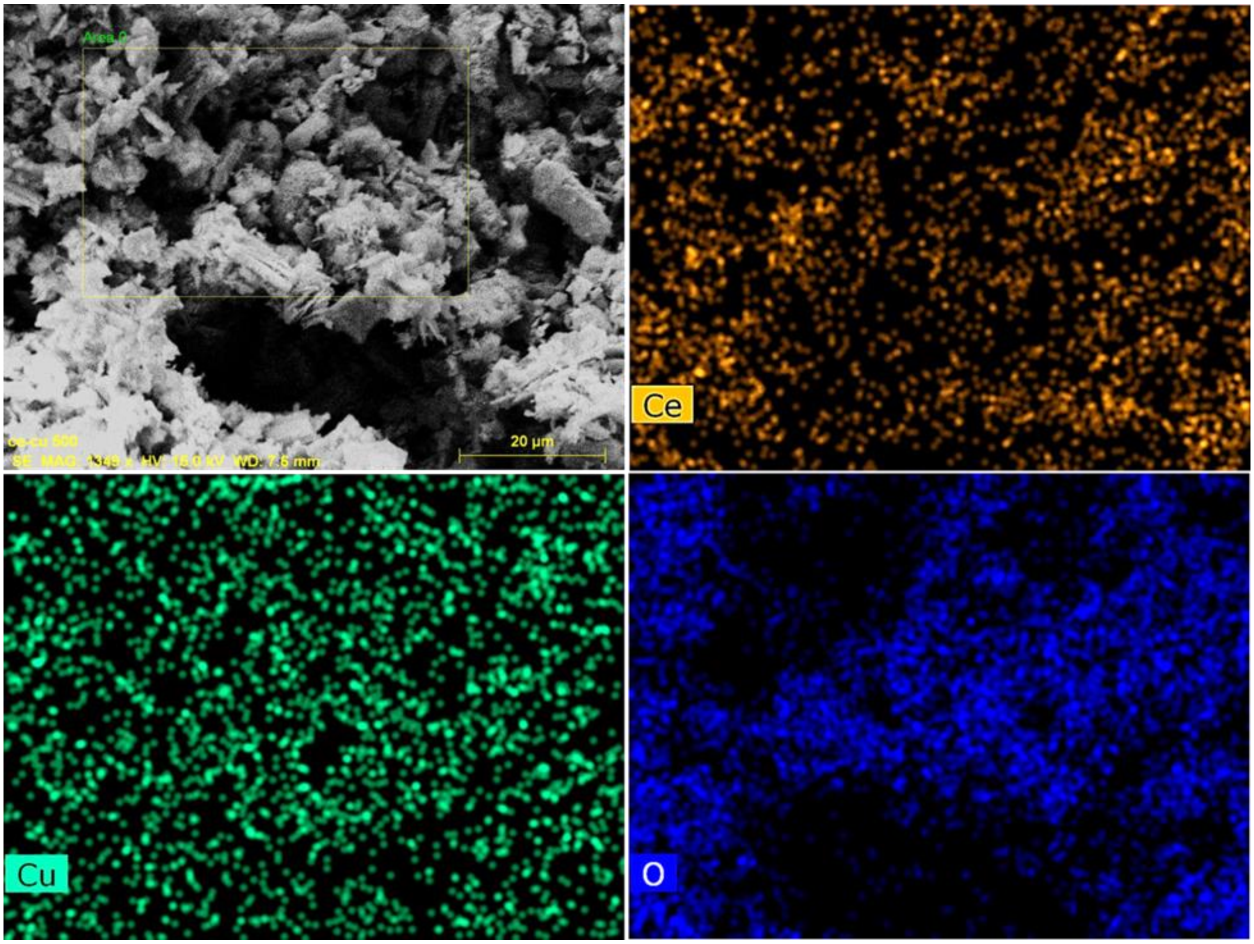

Figure S3: FESEM EDX mapping of CeO_2/CuO composite.

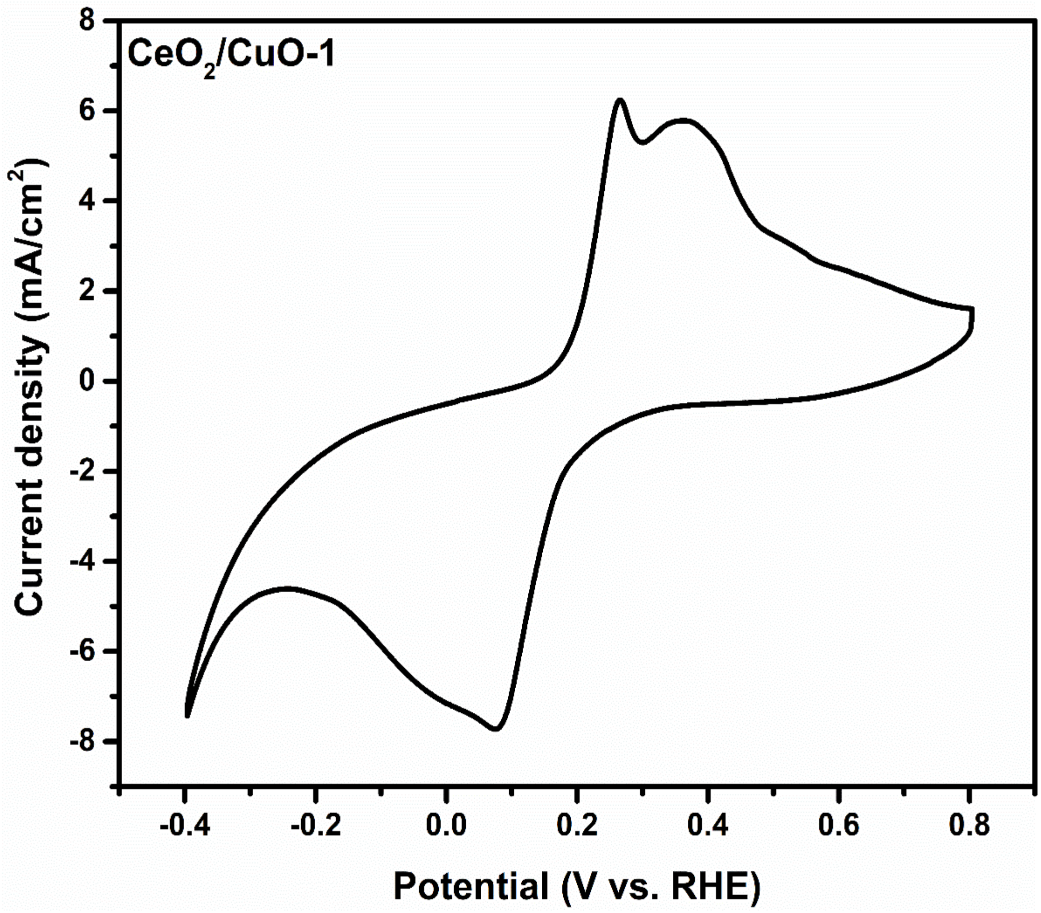

Figure S4: Cyclic voltammetry of CeO_2/CuO-1 at scan rate of 40 mVs^{-1}.

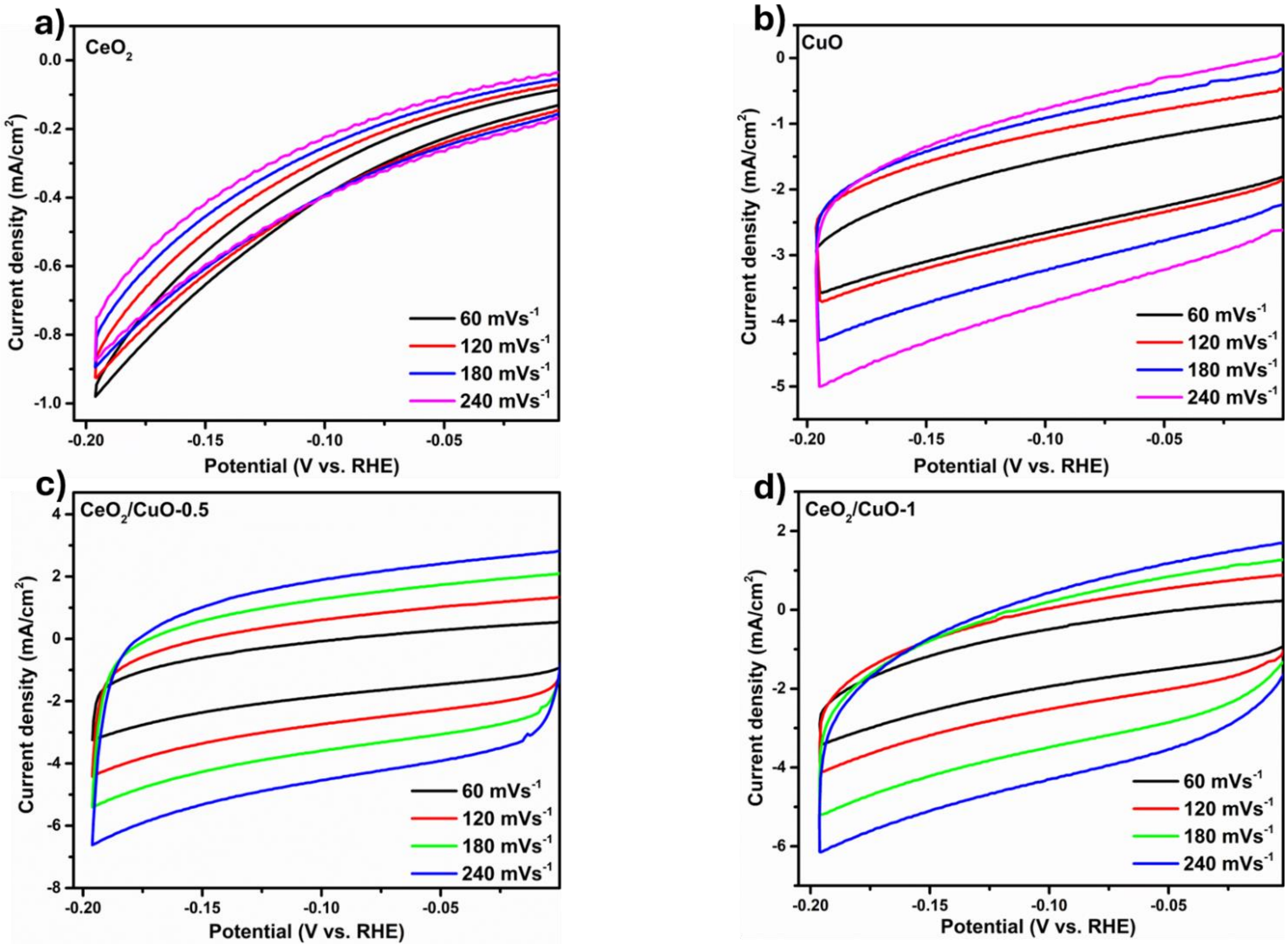

Figure S5: Cyclic voltammetry analysis in non-faradaic region at different scan rates for electrochemical double layer measurement in 1M H_2SO_4 for(a) CeO_2, (b) CuO, (c) CeO_2/CuO-0.5, and (d) CeO_2/CuO-1.

At the center of the potential range in the CV of each sample, the difference in current density (ΔJ) between anodic (Ja) and cathodic (Jc) current densities was measured for each scan rate. To calculate the double-layer capacitance (Cdl), a graph between ΔJ and scan rates (V) was plotted. The slope of the linear fitting graph was applied to determine the C_{dl} value by the use of the equation $C_{dl} = \frac{\Delta J}{V}$. Another equation ($ECSA = \frac{Cdl}{Cs}$) was applied to estimate the ECSA of the prepared samples. Where C_s is standard specific capacitance (C_s), and its value is 0.004 mF/cm^2 for a catalyst having a surface area 1cm^2 [1].

Table S1: Electrochemical double-layer capacitance (C_{dl}) and electrochemically active surface area (ECSA) of prepared samples.

**Samples**	**C_{dl} (mF/cm^2)**	**ECSA (cm^2)**
CeO_2	0.5	12.5
CuO	10.5	262.5
CeO_2/CuO-0.5	28.5	712.5
CeO_2/CuO-1	18.7	467.5

Table S2: R_{ct} and C_{dl} values obtained by fitting EIS plot for different samples.

**Samples**	**Resistant (R_{ct})**	**C_{dl}**
CeO_2	0.4562 ohm	$0.1146x10^{-3}$ F
CuO	0.3902 ohm	$0.187x10^{-3}$ F
CeO_2/CuO-0.5	0.1148 ohm	$0.343x10^{-3}$ F
CeO_2/CuO-1	0.2861ohm	$0.274x10^{-3}$ F

Table S3: Comparison of CuO/CeO_2-based materials with reference to our work

Materials	Synthesis method	Electrolyt e	Overpotential at 10 mA/cm^2 (mV vs. RHE)	Ref.
CeO_2/Ni-TMO	Electrodeposition/annealing	1M KOH	93	[2]
CuO/ZnO	Chemical reduction	1M KOH	358	[3]
CeO_2/RuO_2	Hydrothermal/annealing	0.5 H_2SO_4	120	[4]
$CuWO_4$-CuO	Hydrothermal/annealing	1M KOH	121	[5]
CeO_2@ (Ni, Co)$_2$P/NF	Hydrothermal/Electrodeposition	1M KOH	58	[6]
CuO@NH_2-UiO-66	Solvothermal	1M KOH	166	[7]
CeO_2/CuO	Hydrothermal	1M H_2SO_4	98	**Present work**

References

[1] S. De, S. Roy, G. C. Nayak, *Mater. Today Nano* **2023**, *22*, 100337.

[2] X. Long, H. Lin, D. Zhou, Y. An, S. Yang, *ACS Energy Lett.* **2018**, *3*, 290.

[3] U. Younas, F. Mobeen, A. Saleem, F. Ali, M. Al Huwayz, A. Ashraf, A. Ahmad, N. Alwadai, M. Pervaiz, M. Iqbal, *Ceram. Int.* **2024**, *50*, 30570.

[4] Y. Wu, R. Yao, K. Zhang, Q. Zhao, J. Li, G. Liu, *Chem. Eng. J.* **2024**, *479*, 147939.

[5] X. Chen, X. Li, Z. Chen, B. Wu, Z. Jia, F. Gong, *J. Phys. Chem. Solids* **2024**, *192*, 112079.

[6] H. Sun, B. Jin, S. Cao, J. Zhang, T. Li, X. Liu, G. Liu, J. Li, *J. Electroanal. Chem.* **2023**, *943*, 117591.

[7] M. Fiaz, M. Athar, *Catal. Letters* **2020**, *150*, 3314.
